# Reply to Yang et al.: Global warming and black carbon simultaneously lead to glacier mass decline over the southeastern Tibetan Plateau

**DOI:** 10.1073/pnas.2301467120

**Published:** 2023-03-13

**Authors:** Achille Jouberton, Evan S. Miles, Thomas E. Shaw, Michael McCarthy, Stefan Fugger, Francesca Pellicciotti

**Affiliations:** ^a^Swiss Federal Institute for Forest, Snow and Landscape Research, 8903 Zürich, Switzerland; ^b^Institute of Environmental Engineering, 8049 ETH Zürich, Switzerland; ^c^British Antarctic Survey, Natural Environment Research Council, Cambridge CB3 0ET, UK

In our study (1) we reconstructed the last 45 y of glacial evolution in the Southeastern Tibetan Plateau and revealed that decadal changes in spring and monsoon precipitation have respectively mitigated and accentuated glacier mass losses. The mesoscale perspective of changes to atmospheric circulation and moisture supply described by Yang et al. (2) brings a timely explanation to the decrease in monsoon precipitation noticed by both studies since the early 2000s.

**Fig. 1.**
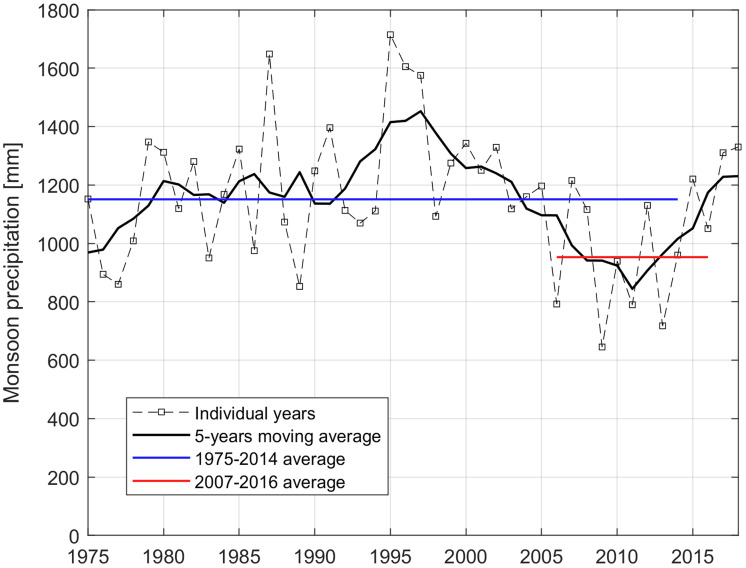
Monsoon precipitation averaged over the Parlung No. 4 catchment, reconstructed since 1975 by Jouberton et al. (1).

**Fig. 2.**
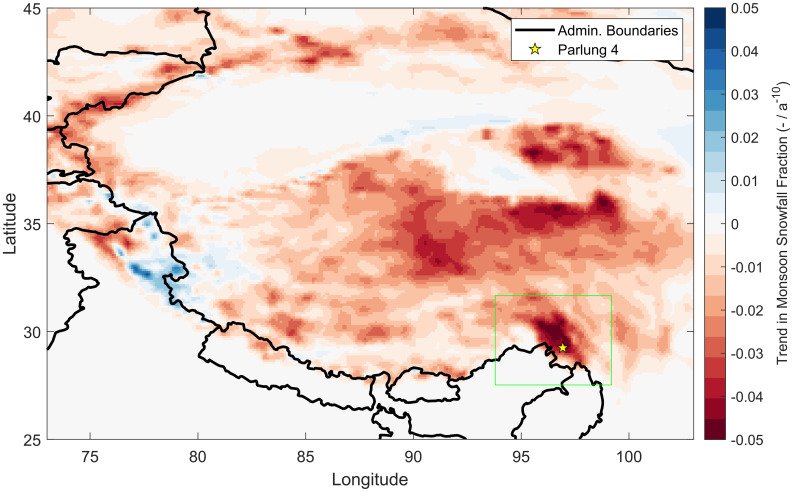
Annual trends in monsoon snowfall ratio over the period 1981 to 2020, derived from ERA5-Land reanalysis data. This figure was adapted from Jouberton et al. (1) (figure S24).

Variations in monsoon moisture supply are a crucial physical process to disentangle, and isolating the influence of atmospheric black carbon (3) is a welcome contribution. The monsoonal moisture supply reductions noted by Yang et al. (3) are evident in our own analyses at Parlung No. 4 Glacier in the Southeastern Tibetan Plateau (Fig. 1), which suggests a reduction in monsoon precipitation of 121 mm for the 2000 to 2018 period as compared to the 1974 to 1999 period. Our results indicate that this decrease in monsoon precipitation led to a 97 mm decrease in monsoon snowfall, representing ~6% of the annual glacier mass accumulation. We performed the same analysis for the periods 2007 to 2016 and 1974 to 2014 considered by Yang et al. (3) and found that monsoon (solid) precipitation decreased by (151) 198 mm (Fig. 1). This represents 9.8% of the annual glacier mass accumulation, consistent with the result of Yang et al. (2). The absolute amounts in precipitation change and glacier mass gain change, however, are different from those of Yang et al. (2; figure 2), which could be due to i) the differences in spatial scale considered for the two analyses (Parlung No. 4 catchment vs. the Southeastern Tibetan Plateau region), ii) differences in annual glacier mass accumulation estimated using different glaciological models and precipitation forcings, and iii) additional processes leading to the monsoon precipitation deficit observed at Parlung No. 4 glacier other than black carbon [e.g., Roxy et al. (4) provided evidence of weakening of the South Asian monsoon due to the Indian Ocean warming].

While the study of Yang et al. (3) demonstrated that the black carbon–related decrease in monsoon precipitation and associated glacier mass loss is especially relevant in the Himalayas, the ERA5-Land trends in monsoon solid precipitation [Jouberton et al. (1); figure S24 and Fig. 2] revealed that the decrease of snowfall due to phase change is particularly strong in the Southeastern Tibetan Plateau. The study of Dong and Ming (5) also demonstrates that recent warming has made rainfall the dominant form of precipitation in High Mountain Asia since the 1990s. Summer and spring–summer accumulation glaciers are especially susceptible to summertime warming (6), and this sensitivity is in part due to precipitation phase changes and nonlinear effects of snowfall decrease on the glacier mass balance through albedo change (7).

The rapid decline of glaciers in the Southeastern Tibetan Plateau threatens the sustainability of its water resources, and understanding these two complementary drivers will help to design mitigation measures and refine projections of glacier and hydrological changes.
